# Congo red dye degradation using Fe-containing mineral as a reactive material derived from waste foundry dust

**DOI:** 10.1007/s11356-024-33064-9

**Published:** 2024-03-28

**Authors:** Hyunsoo Kim, Chulhyun Park, Nagchoul Choi, Kanghee Cho

**Affiliations:** 1 Department of Energy and Resource Engineering, Chosun University, Gwang-Ju, 61452 Korea; 2 Research Institute of Agriculture and Life Sciences, Seoul National University, Seoul, 08826 Korea

**Keywords:** Waste foundry dust, Fe dissolution, Hydroxyl radicals, Fenton oxidation, Congo red

## Abstract

This study investigated the applicability of industrial waste. The high affinity of Fe-based products is widely used for industrial effluents because of their capability to oxidize contaminants. Waste foundry dust (WFD) is an Fe oxide that has been investigated as a potential reactive material that causes the generation of reactive oxidants. We aimed to investigate the physicochemical properties of WFD and the feasibility in the Fenton oxidation process. The WFD was used as a catalyst for removing Congo red (CR), to evaluate the generation of •OH and dissolution of Fe during the oxidation process. The linkage of •OH generation by WFD with eluted Fe(II) through the Fe dissolution was found. The Fenton oxidation reaction, CR degradation was affected by H_2_O_2_ concentration, initial pH, WFD dosage, initial CR concentration, and coexisting anions. The CR degradation efficiency increased with an increase in H_2_O_2_ concentration and WFD dosage. In addition, chloride and sulfate in solution promoted CR degradation, whereas carbonate had a negative effect on the Fenton oxidation process. The elution of Fe promotes CR degradation, over three reuse cycles, the degradation performance of the CR decreased from 100 to 81.1%. For the Fenton oxidation process, •OH generation is linked to Fe redox cycling, the surface passivation and Fe complexes interrupted the release of reactive oxidants, which resulted in the degradation of the CR decreased. This study proposed that WFD can serve as catalysts for the removal of CR.

**Supplementary Information:**

The online version contains supplementary material available at 10.1007/s11356-024-33064-9.

## Introduction

The wastewater effluent from the textile industry, which uses toxic chemicals such as dyestuffs, contains various chromophoric groups that exhibit adverse effects on the environment and human health (Oladoye et al. 2022; Sakkas et al. 2010). The excessive use of these dyes is becoming a severe environmental problem and major global concern. Congo red (CR) is an anionic diazo dye (-N = N =). It is a benzidine-based dye with a stable structure and one of the most frequently used secondary diazo dye (Singh et al. 2016; Xiang et al. 2022). Benzidine is a toxic and carcinogenic metabolite. Therefore, wastewater containing mixtures of organic compounds should be treated appropriately before being discharged into water bodies (Bhaumik et al. 2015; Maruthupandy et al. 2022).

In the past few decades, chemical and physical decolorization techniques including chemical oxidation and adsorption have been widely used to remove organic pollutants from industrial wastewater (Chu et al. 2020; Chen et al. 2019a; Kang et al. 2021; Moon et al. 2023). Advanced oxidation processes (AOPs) based on the generation of the hydroxyl radical (•OH) have been extensively studied and used because of their effectiveness for pollutant removal (Chu et al. 2021a; Baba et al 2015; Maezono et al. 2011). The Fenton process relies on •OH, which plays an active role in the degradation of pollutants. Fe-based materials are highly reactive and can oxidize organic pollutants. These have attracted considerable attention as remarkable catalysts (Harada et al. 2016; Liu et al. 2020; Cai et al. 2022). It is known that •OH generation is linked to Fe redox cycling. These reciprocally affect each other through the oxidation of Fe(II) and reduction of Fe(III). Studies have demonstrated that the oxidation of Fe(II) to Fe(III) is significantly faster than the conversion of Fe(III) to Fe(II) (You et al. 2022; Fujioka et al. 2016; Dai et al. 2022; Jung et al. 2022) In general, Fe-based materials in the H_2_O_2_ system has higher reaction rates for Fe(II) (40–80 M^−1^ s^−1^) than for Fe(III) (9.1 $\times$ 10^−7^ M^−1^ s^−1^) (Eqs. (1)–(3)). Previous studies have reported that accumulated Fe(III) can facilitate the generation of Fe(OH)_3_ and thus undergo an increase in pH. It finally forms inactivated Fe sludge (Eqs. (4)–(5)) (Zou et al. 2014; Yang et al. 2020; Wei et al. 2021):

1 $${\text{Fe}}({\text{II}})+{{\text{H}}}_{2}{{\text{O}}}_{2}\to {\text{Fe}}({\text{III}})+\cdot {\text{OH}}+{{\text{OH}}}^{-}$$

2 $${\text{Fe}}({\text{III}})+{{\text{H}}}_{2}{{\text{O}}}_{2}\to {\text{Fe}}({\text{III}})+{{\text{HO}}}_{2}\cdot +{{\text{H}}}^{+}$$

3 $${\text{Fe}}({\text{III}})+{{\text{HO}}}_{2}\cdot \to {\text{Fe}}({\text{II}})+{{\text{O}}}_{2}\cdot +{{\text{H}}}^{+}$$

4 $${\text{Fe}}({\text{II}})+{2{\text{OH}}}^{-}\to {{\text{Fe}}({\text{OH}})}_{2}\to {\text{FeO}}+{{\text{H}}}_{2}{\text{O}}$$

5 $$3{\text{Fe}}({\text{III}})+{6{\text{OH}}}^{-}\to {2{\text{Fe}}({\text{OH}})}_{3}\to {{\text{Fe}}}_{2}{{\text{O}}}_{3}+{3{\text{H}}}_{2}{\text{O}}$$

To overcome these problems, many alternatives to Fe-based materials have been developed. These drawbacks can be overcome by the addition of metal sulfides as co-catalysts (He et al. 2023; Xing et al. 2018; Cheng et al 2023). According to the literature, the addition of sulfide minerals such as molybdenum sulfide (MoS_2_) and tungsten disulfide (WS_2_) can improve the low cycling efficiency of Fe catalysts. For example, WS_2_ can enhance the rate-limiting step of Eq. (2) because of the exposed W(IV) active sites on its surface. He et al. (2023) demonstrated that the oxidation of W(IV) and unsaturated S on the surface of WS_2_ contributed significantly to the reduction of Fe(III) to Fe(II).

A recent literature survey revealed that other inexpensive materials have been investigated because of various heterogeneous Fenton processes (Xiao et al. 2021; Cheng et al. 2021; Hu et al. 2018; Nasuha et al. 2021). In addition, industrial slags are the most popular promoters because these reduce the associated operational costs. Previous studies have reported that the application of the waste slag such as Fe oxides (α-Fe_2_O_3_, γ- Fe_2_O_3_, Fe_2_O_3_, Fe^0^/Fe_2_O_3_, α-FeOOH) from industrial activities is used for pollutant removal. In addition, it has been reported as a remarkable catalyst because of its high activity and low cost.

Waste foundry dust (WFD) is a byproduct of metal casting in foundries. It consists of fine particles formed during high-temperature thermal treatments. Additionally, WFD has attracted increasing attention because of their high Fe content, high surface reactivity, and low toxicity (Rha and Jo 2021; Kim et al. 2022; Choi et al. 2012; Yoon et al 2023). Notwithstanding its potential, many researchers have recycled WFD as a raw material to produce bricks or concrete. It has the potential to be used as an alternative material in the field of wastewater treatment for its capability to generate active radicals such as •OH. A previous study verified that WFD can be used for removing As(III) and Cr(II). WFD oxidation produces electrons. This results in oxidation to As(V) and reduction to Cr(III), followed by adsorption and co-precipitation, respectively (Rha and Jo 2021). Waste cast iron (WCI) is used as a functional material to manufacture ceramic membranes for removing Se(IV) from wastewater (Yoon et al 2023). In addition, WFD-based alginate beads have been used successfully as supports for synthesizing functionalized adsorbents that can be separated conveniently after reaction (Kim et al. 2022). Most previous studies focused on inexpensive adsorbents or support materials based on WFD. Notwithstanding these potential materials, the demand for byproducts is low. This causes environmental problems. Therefore, more work is required to increase the application of byproducts. In this study, we investigated the physicochemical properties of WFD and its feasibility for Fenton oxidation. The specific objectives of this study were to evaluate the generation of •OH and dissolution of Fe during the oxidation process. Subsequently, the effects of various factors (including the H_2_O_2_ concentration, WFD dosage, initial pH, and CR concentration) and those of coexisting chlorides, carbonates, and sulfate on the degradation of CR were examined based on the WFD–H_2_O_2_ system.

## Materials and methods

### Materials and chemicals

The WFD samples used in this study were obtained from a foundry in Incheon, South Korea. CR (C_32_H_22_N_6_Na_2_O_6_S_2_) was purchased from Samchun Chemicals. Hydrogen peroxide (H_2_O_2_, 30%), hydrochloric acid (35%), sodium hydroxide (98%), sodium chloride (NaCl), sodium carbonate (Na_2_CO_3_), sodium sulfate (Na_2_SO_4_), and sodium acetate were purchased from Duksan Pure Chemicals. 1,10-Phenantroline and hydroxylamine hydrochloride were obtained from Daejung Chemicals. Disodium telephthalate (TA, > 99%) was purchased from Alfa Aesar (USA). 2-Hydroxyterephthalate (TPOH 97%) was purchased from Sigma–Aldrich. The deionized water used in this experiment was purified using an EXL5 system (Vivagen Co. Ltd., Korea).

### WFD characterization

X-ray fluorescence (XRF; S4 PIONEER, Bruker AXS, Germany) was used to determine the chemical composition of the WFD (Table 1). The WFD was mainly composed of Si (11.9 wt%) and Fe (28.0 wt%). It had small amounts of Ca (1.0 wt%). Its magnetic properties were observed at room temperature using a vibrating-sample magnetometer (VSM; LakeShore 7407-S, Lake Shore Cryotronics, Inc., USA). The saturation magnetization of the WFD was 23.3 emu/g. The specific surface area of the WFD was determined to be 1.06 m^2^ g^−1^ using the BET-N_2_ method (BELSORP-max, BEL Japan Inc., Japan). The average particle size of the WFD was determined using a particle size analyzer (Mastersizer 2000, Malvern Panalytical Ltd., UK). The median particle size (D50) was 24.9 µm.

**Table 1:** Chemical composition of WFD

Elements	Fe	Si	Zr	Al	Cu
Content (wt%)	28.0	11.9	7.48	3.86	1.96
Elements	S	Zn	K	Ca	Mn
Content (wt%)	1.62	1.60	1.23	1.03	0.91

Heavy metals were leached from WFD using the modified Korean standard leaching test (KSLT). Briefly, batch tests were conducted with solid-to-liquid ratios of 10 g/L. Their pH was maintained in the range of three to nine by adjusting with a 0.1 M HCl and NaOH solution. After the reaction, the pH of the suspension was determined using a pH meter. The suspensions were analyzed for heavy metals using ICP-OES (Perkin Elmer Optima Model 5300DV, PerkinElmer Inc., USA).

### Fe dissolution and OH radical generation by WFD

To evaluate the generation of hydroxyl radical (•OH), WFD was added to a beaker containing an appropriate amount of deionized water. Batch experiments were conducted at liquid-to-solid (L/S) ratios of 1, 5, and 10 for 30 and 60 min at 120 rpm. The •OH in each sample was determined by a fluorescence spectrophotometer (FluoroMate FS-2, SCINCO, Korea). To detect the •OH generated during the processes, disodium terephthalate (TP) was used as a probe molecule. The reaction between TP and •OH produced 2-hydroxyterephthalate (TPOH) (Soyluoglu et al. 2021). The fluorescence intensity was measured using a fluorescence spectrophotometer from the fluorescence spectra of TPOH, which was excited at 315 nm to emit fluorescence at 425 nm.

The Fe-eluted solution in the reaction solution was then measured. The concentrations of Fe(II) and total Fe ions were determined using a modified 1,10-phenantroline method (Chen et al. 2019b). To detect dissolved Fe (II), 0.5 mL of 1,10-phenantroline solution (5 mM), 0.5 mL of sodium acetate (1 M), and 0.5 mL of deionized water were added sequentially to 0.5 mL of a sample solution. Similarly, 0.5 mL of 1,10-phenanthroline solution (5 mM), 0.5 mL of deionized water, 0.5 mL of sodium acetate (1 M), and 0.5 mL of hydroxylamine hydrochloride were added sequentially to 0.5 mL of the sample solution. The Fe(II)-1,10-phenanthroline complex was analyzed using a UV–vis spectrophotometer (OPTIZEN POP, KLAB, Korea) at a detection wavelength of 510 nm (Fig. S1).

### Congo red removal experiments

The degradation experiments were performed in a 50 mL vial at room temperature. The experiment was conducted at different WFD concentrations (10–100 mg/L), dosages (1–2 g/L), and pH values (4–10) with constant stirring at 120 rpm. Different concentrations (10–100 mM) of hydrogen peroxide were introduced to aqueous solutions to initiate the reactions. The pH of the solution was adjusted using a solution of HCl or NaOH. Experiments on the effect of coexisting anions on the CR degradation performance were confirmed using NaCl, Na_2_CO_3_, and Na_2_SO_4_ at 1.17, 3.42, 8.56, and 17.11 mM, with a CR concentration of 100 mg/L. For the recycling experiments, the WFD was washed with deionized water and reused. The concentration of CR was analyzed using a UV–vis spectrophotometer at a detection wavelength of 498 nm (Fig. S2). The experiments were performed in duplicate, and the results were expressed as mean values.

### Characterization methods

The WFD was analyzed using X-ray diffraction (XRD, X’Pert Pro MRD, PANalytical, Netherlands). Cu Kα X-rays were used at an acceleration voltage of 40 kV and a current of 30 mA. The morphology and surface structure of the samples were analyzed using field-emission scanning electron microscopy (FE-SEM, S4800, Hitachi, Tokyo, Japan) with energy-dispersive X-ray spectroscopy (EDS, ISIS310, Jeol, Japan). The chemical bonding and elements were studied using X-ray photoelectron spectroscopy (XPS, Thermo Scientific K-Alpha spectrometer) with an X-ray source of Al Kα radiation. The surface functional groups of WFD were verified using a Fourier-transform infrared (FTIR) spectrometer (Nicolet 6700, Thermo Scientific, USA). The CR intermediate products were identified by LC–MS (Qtrap 4500, AB SCIEX, USA) coupled with MS. Measurement conditions of LC–MS were as follows: column, Agilent Poroshell 120 EC-C18 (2.1 × 100 mm, 2.7 µm); mobile phase, 0.1% formic acid in water and 0.1% formic acid in acetonitrile; flow rate, 0.3 mL/min.

## Results and discussion

### Characterization of WFD

**Fig. 1:**
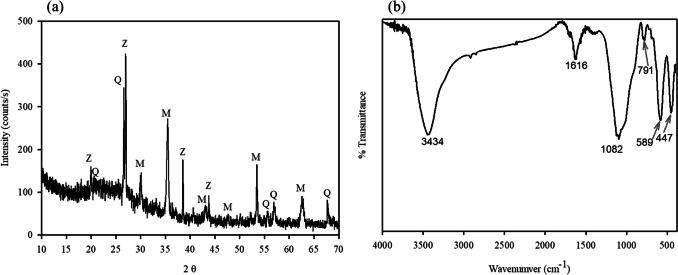
**a** XRD pattern and **b** FTIR of WFD

**Fig. 2:**
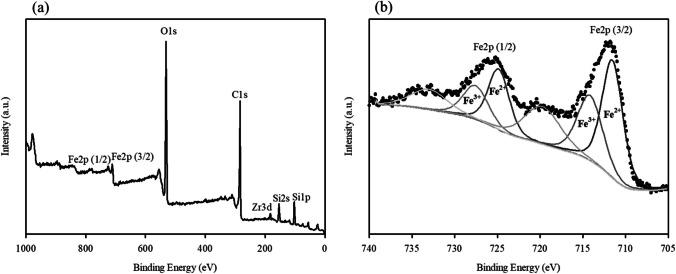
XPS spectra of WFD. **a** Wide scan. **b** High-resolution scan of Fe 2p

The FESEM images show that the morphology of the WFD consisted of spherical particles with a smooth surface. The EDS pattern showed that characteristics of WFD were primarily composed of Fe (41.8 wt%), O (31.0 wt%), Si (15.0 wt%), and Zr (12.0 wt%). It is evident that Fe was the major element. The sample was investigated via XRD. This revealed that it consisted of magnetite, quartz, and zirconium oxide (Fig. 1a). The FT-IR spectrum (589 cm^−1^) also matches well with the stretching vibration of the Fe–O bond of magnetite (Fig. 1b) (Radu et al. 2017; Chu et al. 2021b). In addition, it was verified that the peaks at 1082, 791, and 447 cm^−1^ correspond to Si–O vibrations (Rha and Jo 2021; Kim et al. 2022). The XPS spectra of WFD are presented in Fig. 2. The C 1 s, O 1 s, Si 2p, Fe 2p, and Zr 3d peaks are visible in the spectra. In the high-resolution scan of the Fe 2P spectra, the binding energy of 711.5 eV could be attributed to magnetite contain Fe(II). Meanwhile, the peak at 714.1 eV corresponds to Fe(III) oxy-hydroxide (Rha and Jo 2021; Chu et al. 2021b).

To evaluate the leaching of heavy metals, experiments were conducted at different initial pH values (Table 2). At pH 3.0 and 5.0, the solution pH increased. This may have been owing to the release of Ca from the WFD. These results indicate that the pH of the solution increased after the reaction (in the yield of reactive oxidants and in the production of Fe). This reduced the performance. However, at pH 7.0 and 9.0, the release of Ca had no effect on the pH. In addition, Fe was below the detection limit (< 0.1 mg/L) for all the pH values used in this study. To verify the variations in the Fe–O peak after the leaching reaction at different initial pH values, the WFD was verified by FT-IR (Fig. S3). There was no change in the Fe–O peaks before and after the reaction. This indicated that WFD could be used as a reactive material to remove aquatic pollutants.

**Table 2:** Heavy metals leaching of WFD at various pH according to the modified KSLT (unit, mg/L)

Sample	Final pH	As	Ca	Cd	Cu	Fe	K	Mn	Pb
pH3	5.24	N.D	258.98	N.D	1.90	N.D	38.13	32.48	N.D
pH5	5.58	N.D	202.73	N.D	1.14	N.D	23.66	17.70	N.D
pH7	5.67	N.D	199.75	N.D	0.88	N.D	23.45	17.21	N.D
pH9	5.68	N.D	217.35	N.D	1.05	N.D	28.07	23.01	N.D

### Fe dissolution and OH radical generation by WFD

**Fig. 3:**
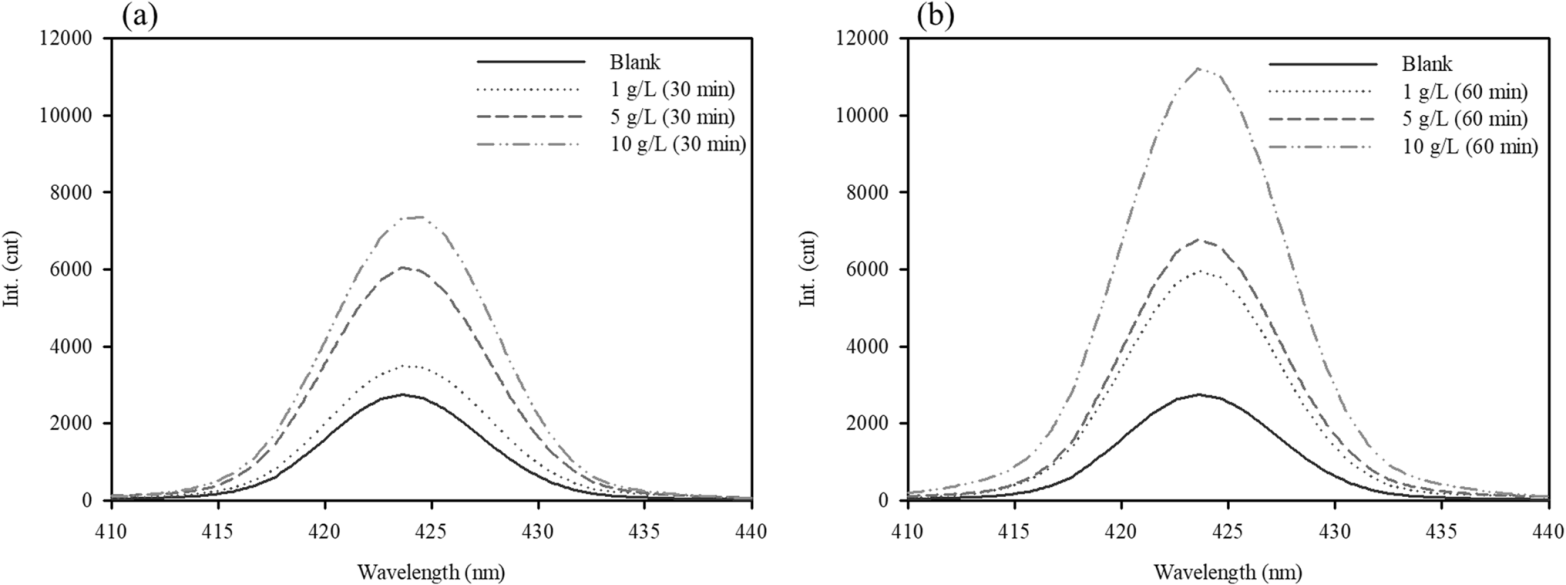
Photoluminescence spectra of WFD under different WFD dosage: **a** 30 min and **b** 60 min

**Fig. 4:**
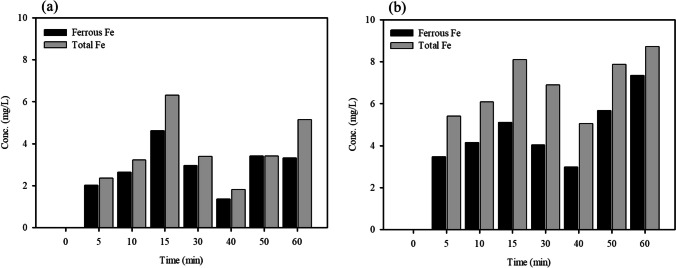
The concentration of ferrous Fe and total Fe in **a** WFD and **b** WFD-H_2_O_2_. Conditions: WFD = 1%, H_2_O_2_ = 100 mM

In general, •OH generation is closely linked to Fe elution. The generation of •OH and dissolution of Fe in the solution were evaluated. Figure 3 shows the variations in fluorescence intensity at different time intervals. It is evident that the intensity increased gradually with an increase in the reaction time. The increase in the intensity of •OH when WFD dosage was increased could be attributed to an increase in the amount of emitted electron, which promotes the generation of •OH (Xing et al. 2018). It has been established that the Fe elution is linked with the generation of •OH in solution. To understand this, the formation of Fe(II) and total soluble Fe were measured during the initial 60 min (Fig. 4). It was observed that Fe(II) was released straightforwardly, with an initial increase in Fe(II) and then a decrease in concentration. Meanwhile, the Fe concentration in the system with H_2_O_2_ was higher than that in the system without it, whereas both types of released Fe showed similar trends. This is attributed to the fact that the consumption of Fe(II) during the Fenton reaction promotes the dissolution of Fe. The •OH generated by the Fenton or Fenton-like reaction can be expressed by the following equations (Eqs. (1) and (2)).

**Fig. 5:**
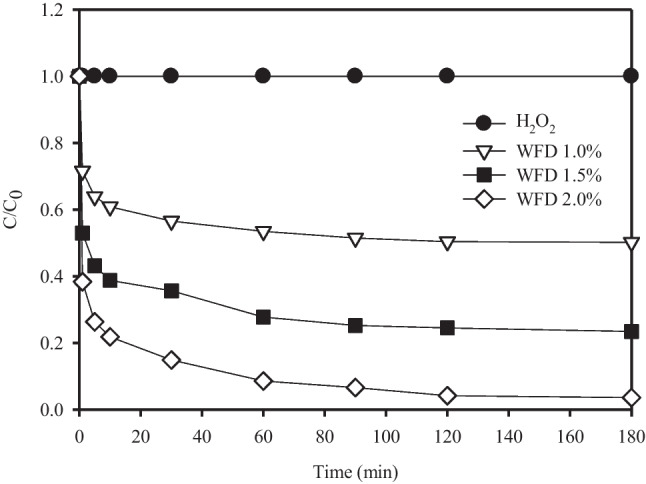
The degradation efficiency of CR by WFD dosage and H_2_O_2_. Conditions: H_2_O_2_ = 100 mM, Congo red concentration = 100 mg/L

As shown in Fig. 5, preliminary experiments with different dosages of WFD at pH 7 were conducted to determine the effect of the WFD dosage. With an increase in the WFD dosage, the removal rate increased. In contrast, H_2_O_2_ did not undergo CR degradation after 180 min. This experimental result suggests that the H_2_O_2_ oxidation alone could hardly degrade CR and did not produce free radicals. It may be represented that Fe(II)/Fe(III) in the WFD can be released and serve as electron donor, which further accelerated the generation of H_2_O_2_, Fenton reaction, and Fenton-like reaction (Ogawa and Kawase 2021; Dai et al. 2022).

### Effects of parameters of Fenton oxidation process on CR removal

#### Effect of initial H_2_O_2_ concentration

**Fig. 6:**
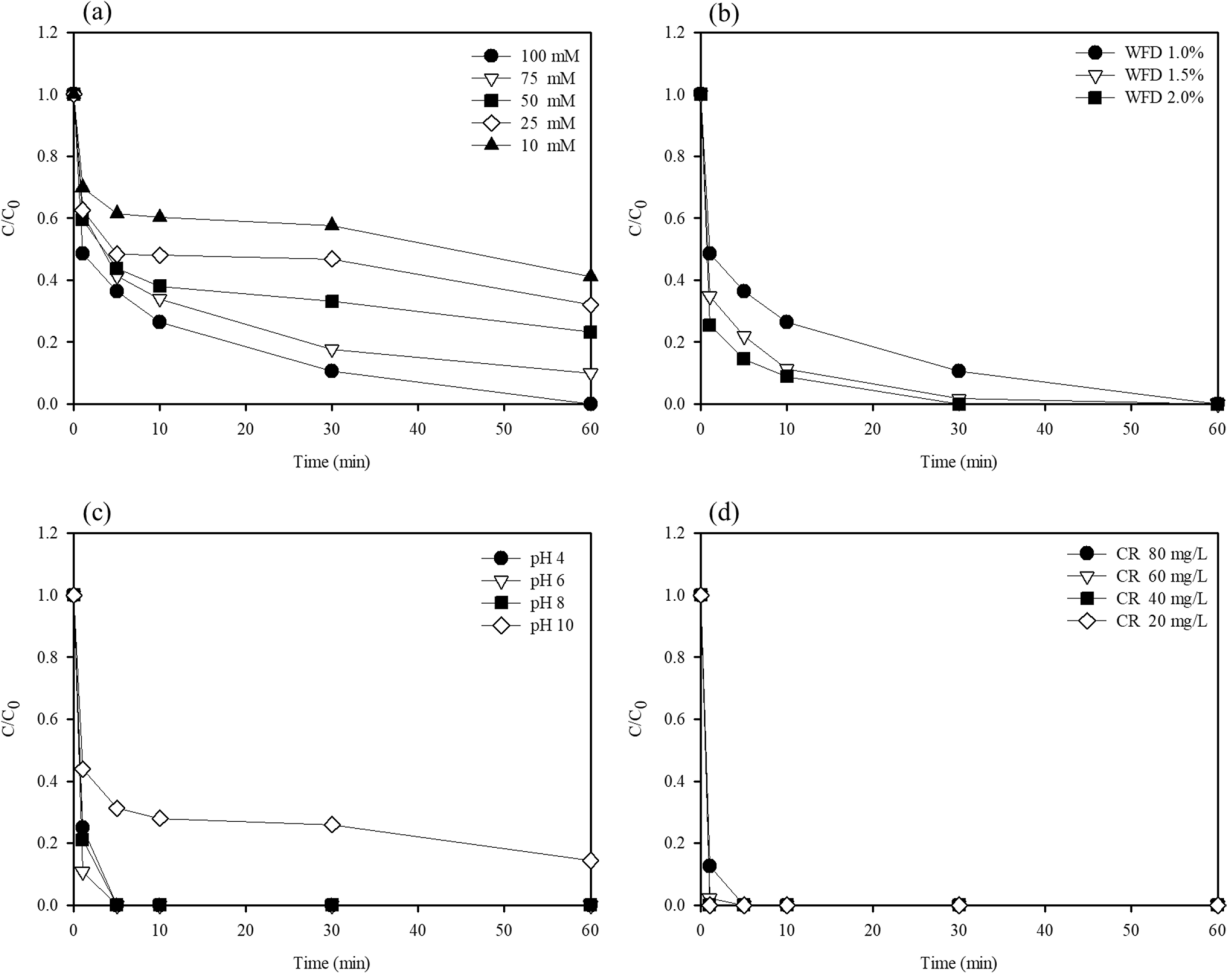
Degradation efficiency in difference reaction systems. **a** Effect of H_2_O_2_ concentration. **b** WFD dosage. **c** Effect of pH. **d** Effect of initial Congo red concentration. Conditions: WFD = 1%, H_2_O_2_ = 100 mM, Congo red concentration = 100 mg/L

The effect of CR degradation on the initial H_2_O_2_ concentration was evaluated. In general, H_2_O_2_ plays a major role as an oxidant in the Fenton oxidation. Figure 6a shows that the CR degradation in the WFD-H_2_O_2_ system tended to increase with an increase in the initial H_2_O_2_ concentration. The CR degradations at 60 min were 58.84%, 68.01%, 76.75%, 89.97%, and 100.00% at H_2_O_2_ concentrations of 10, 25, 50, 75, and 100 mM, respectively. This result indicated that higher H_2_O_2_ concentrations generated more active •OH with no scavenger effects under 100 mM H_2_O_2_, thereby increasing the CR removal rate. However, the excess H_2_O_2_ may cause scavenger effects to produce perhydroxyl radicals (HO_2_⋅) having significantly lower oxidation capabilities than •OH (Eq. (6)). In addition, oxidizing capacity of the formed HO_2_⋅was lower than •OH, which have a negative effect on CR degradation. Meanwhile, for Fe oxides, which is relatively low rate in H_2_O_2_ activation (Yang et al. 2019). For example, compared to the reaction between Cu catalysts and H_2_O_2_, the reaction kinetics of Cu (I)(104 M^−1^ s^−1^) is relatively higher than Fe (II) (63 M^−1^ s^−1^). In this regard, the decomposition of H_2_O_2_ by Fe may lead to relatively low consumption of H_2_O_2_ than Cu (Ghasemi et al. 2021).

6 $${{\text{H}}}_{2}{{\text{O}}}_{2}+\cdot {\text{OH}}\to {{\text{H}}}_{2}{\text{O}}+{{\text{HO}}}_{2}$$

#### Effect of WFD dose

As mentioned above, the WFD can directly affect the CR removal rate at different dosages. As shown in Fig. 6b, the CR degradation performance increased with the dosage, which indicated that the number of active sites for the catalytic degradation of CR increased due to the generation of more •OH as the dose of catalyst increases (Chu et al. 2021b). At a WFD dosage of 2%, the decomposition rate was significantly high, and complete degradation occurred within 30 min. For WFD, on the other hand, the excellent solubility of Fe(II) can inhibit the Fenton process by rapid oxidation of Fe(II) into Fe precipitates (Baba et al. 2015). This occurred because the dosage of WFD was closely linked with the Fe(II) release and •OH generation.

#### Effect of pH

In general, the effect of the initial solution pH is considered an important parameter in Fenton oxidation processes. Figure 6c shows the influence of the initial pH (4, 6, 8, and 10) on the degradation of CR in the WFD-H_2_O_2_ system. Except at an initial pH of 10, the removal efficiencies exhibited similar trends. This is attributed to the fact that the weakened formation of •OH caused by Fe(II) release rate at high pHs and as a result the speciation of Fe(III) towards hydroxide complex species, which were controlled the generation of radicals. Previous studies have reported that the catalytic species and H_2_O_2_ stability are also strongly affected by the pH conditions (Haris et al. 2022).

#### Effect of initial CR concentration

The effects of different initial CR concentrations on the CR removal efficiencies were studied. The results are presented in Fig. 6d. It is evident that the CR degradation efficiency decreased with an increase in the initial concentrations. This occurred because it suppresses the active sites of WFD, which is attributed to the increased number of CR molecules occupying the active sites. Then, the generation of •OH of WFD decreased, which was consistent with previous reports (Rha and Jo 2021).

#### Effect of anions on CR removal

**Fig. 7:**
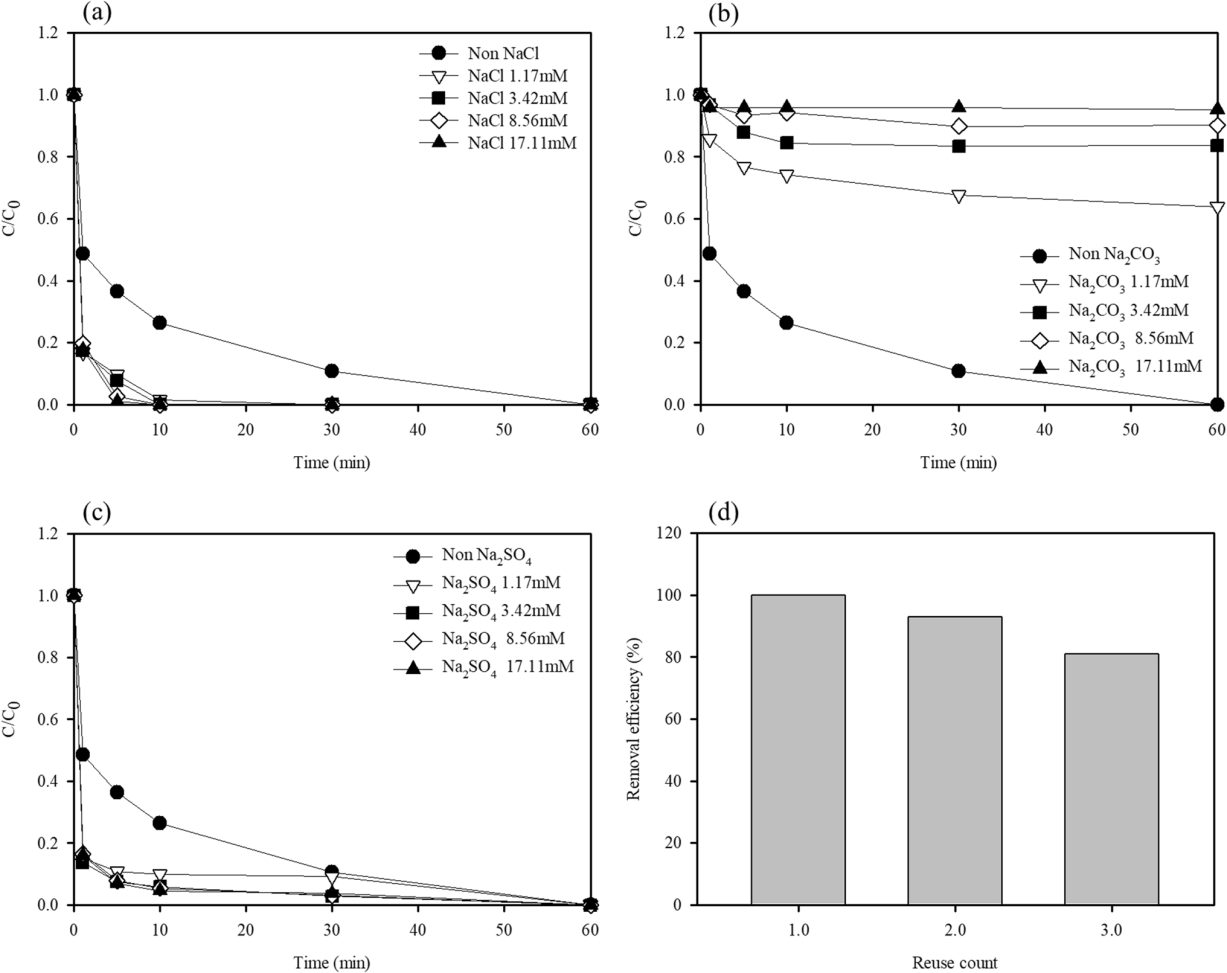
Degradation efficiency in difference reaction systems. **a** Effect of NaCl concentration. **b** Effect of Na_2_CO_3_ concentration. **c** Effect of Effect of Na_2_SO_4_. **d** The recyclability of WFD. Conditions: WFD = 1%, H_2_O_2_ = 100mM, Congo red concentration = 100 mg/L

The effects of the coexisting substances on the CR degradation performance were studied using anions such as chlorides, carbonates, and sulfate. In general, a solution containing anions affects •OH generation and functions as a radical scavenger in the process (Cheng et al. 2023). The effect of coexisting anions in the WFD-H_2_O_2_ system was verified using NaCl, Na_2_CO_3_, and Na_2_SO_4_ (Fig. 7). The CR removal efficiency increased as the chloride and sulfate concentrations increased. This can be attributed to the marginal positive effect on the release of Fe and the improvement in the degradation efficiency of CR. However, the CR degradation deteriorated at all the carbonate concentrations, compared with the scenario without carbonates. Carbonate ions exert negative effects (as scavengers) on the degradation properties of WFD-H_2_O_2_ systems (Chu et al. 2021a). This may be because the hydrolysis of HCO_3_^−^ in the solution causes a reduction in the ⋅HO generation rate and HCO_3_^−^ can scavenge •OH. This could be attributed to the production of peroxymonocarbonate (HCO_4_^−^) and a higher reactivity than that of H_2_O_2_.

7 $${HCO}_{3}^{-}+{{\text{H}}}_{2}{\text{O}}\to {{\text{H}}}_{2}C{{\text{O}}}_{3}+{OH}^{-}$$

8 $${HCO}_{3}^{-}+\cdot {\text{OH}}\to {HCO}_{3}^{\cdot }+{OH}^{-}$$

9 $${CO}_{3}^{2-}+\cdot {\text{OH}}\to {CO}_{3}^{\cdot -}+{OH}^{-}$$

10 $${HCO}_{3}^{-}+{{\text{H}}}_{2}{{\text{O}}}_{2}\to {{\text{H}}}_{2}{\text{O}}+{HCO}_{4}^{-}$$

#### The recyclability and stability of WFD

**Fig. 8:**
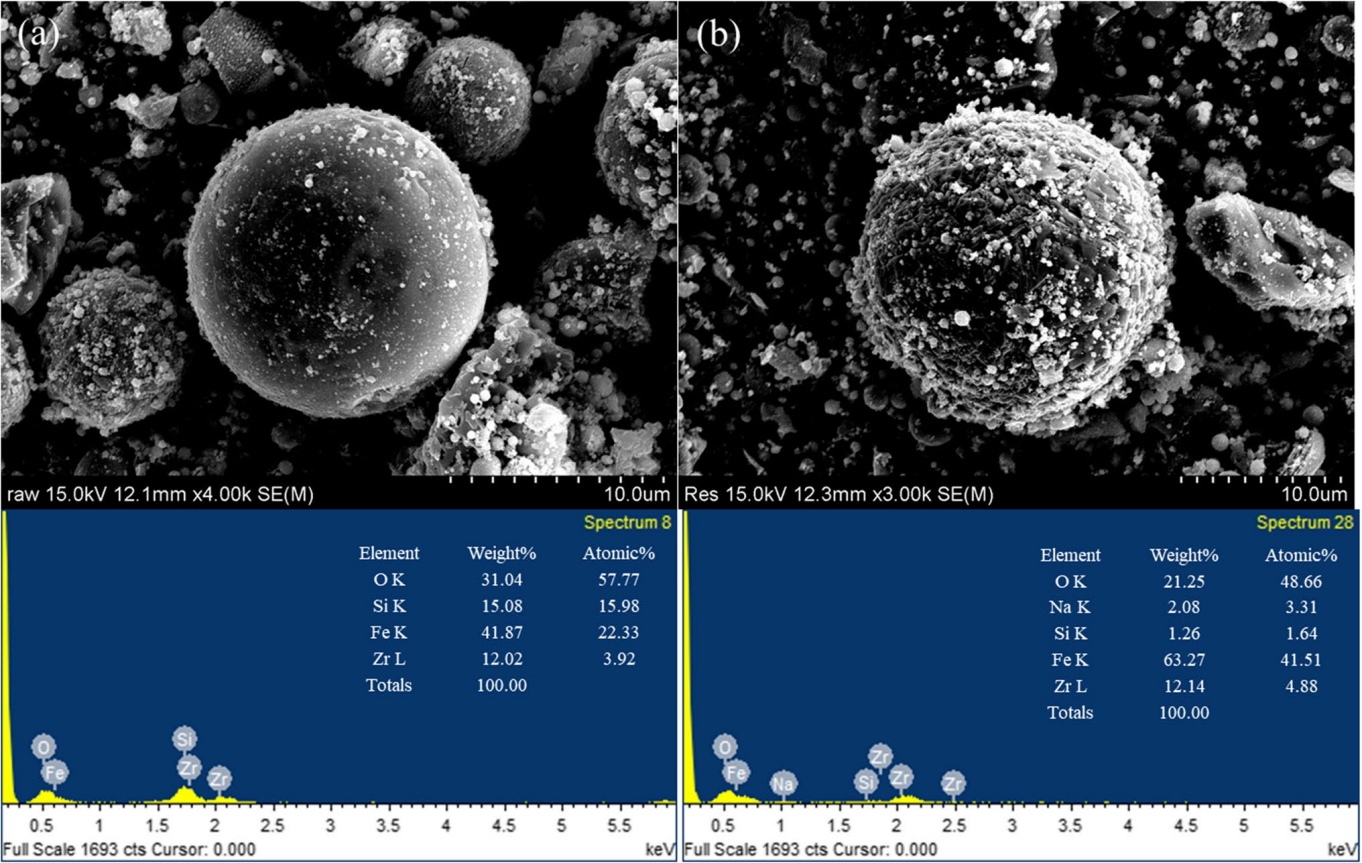
SEM images and EDS analysis of the WFD catalyst **a** before and **b** after CR degradation

Catalyst recyclability is an important factor in the Fenton oxidation. The WFD was separated using a magnet, washed with deionized water, and reused. As shown in Fig. 7d, over three reuse cycles, the degradation performance of the CR decreased from 100 to 81.1%. To verify the stability of the WFD, XPS analysis was performed for comparison with fresh samples. The O 1s and Fe 2p XPS results indicated that the Fenton process did not affect the chemical structure of the WFD. It is evident that the state of the Fe species did not vary in the WFD during multiple uses (Fig. S4). Additionally, the morphological of WFD before and after CR removal was characterized using SED-EDS (Fig. 8). After the reaction, the WFD revealed that the small particles with bound to the surfaces of spherical particle. It was confirmed that small particles are dispersed heterogeneously, which form Fe oxidation products such as oxide or hydroxide (Eqs. 4–5). These results suggest that the formation of Fe oxide layer blocks was involved in the Fenton process, while it enhances the adsorption of CR or its intermediates (Fujioka et al. 2016). Subsequently, the difference in CR degradation performance was due to the reduction of the active sites on the surface of the WFD.

### The reaction mechanisms

**Fig. 9:**
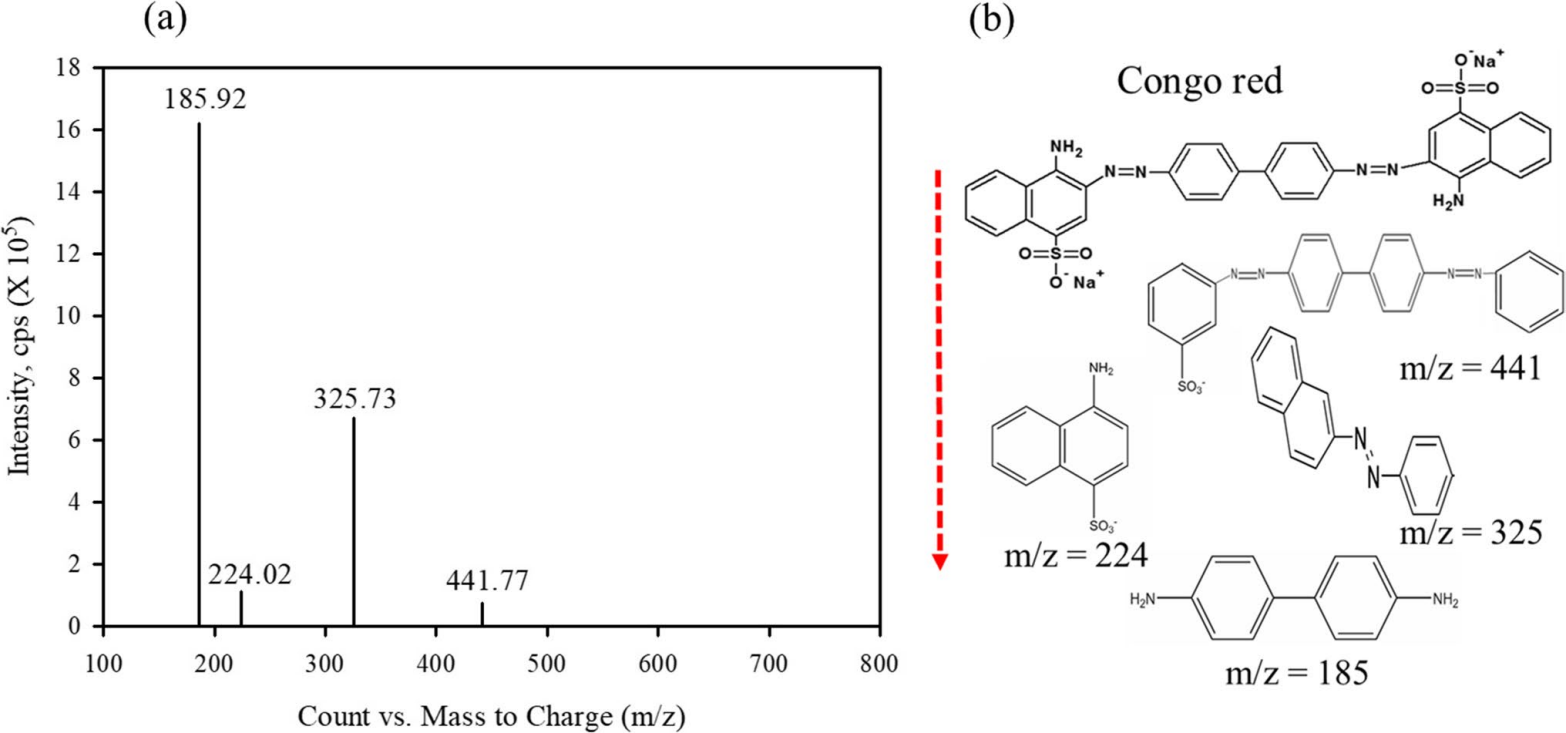
**a** LC–MS spectra of intermediate products resulted from CR degradation. **b** Chemical structure of major decomposition species of CR. Conditions: WFD = 1%, H_2_O_2_ = 100mM, Congo red concentration = 100 mg/L

As mentioned earlier, the oxidation of WFD may be the main source of Fe. Fe is released from the magnetite surface, thereby resulting in the production of electrons. Thereafter, a series of electron transfer occurs, produces •OH acts as reactive oxidants in the system. When H_2_O_2_ was added, Fe(II)/Fe(III) in the WFD can be released, which further accelerated the generation of •OH, and the formation of Fe oxides occurred mainly on the surface. Meanwhile, these results suggest that the surface passivation and Fe complexes interrupted the release of reactive oxidants, which resulted in the mass transfer resistance. CR is an anionic diazo dye (-N = N =), a benzidine-based dye with a stable structure, and one of the most frequently used secondary diazo dye (Singh et al. 2016; Xiang et al. 2022). We identified the intermediated produced in the CR degradation by LC–MS analysis. As shown in Fig. 9, the intermediate products of CR formed in the Fenton oxidation process revealed the species at m/z ratios of 185, 224, 325, and 441. These results suggest that azo bond cleavage (m/z = 441 and 325) and azo bond breaking (m/z = 224 and 185) were attributed to •OH generated by the Fenton oxidation process. For the Fenton process, the -N = N = groups in the CR were broken and formation of amines (-NH_2_) in the CR reduction, while the formation of Fe oxides adsorbed the CR degradation intermediate products by acting as sorbents.

## Conclusions

In this study, we investigated the applicability of WFD to CR degradation. The •OH generation by WFD was examined by focusing on the linkage with Fe dissolution. The linkage of •OH generation by WFD with eluted Fe(II) through the Fe dissolution was found. With increasing WFD dosage, the amount of generated •OH for WFD increased. This confirms the generation of •OH affected by the change in the Fe(II) release. This suggests that Fe(II)/Fe(III) in the WFD can be released, and Fe dissolution can influence pollutant degradation through the generation of •OH. The degradation of CR over time was compared in different processes involving H_2_O_2_ concentration, WFD dosage, and solution pH as well as the coexisting anions. The CR degradation efficiency in the Fenton oxidation process was enhanced with increasing H_2_O_2_ concentration and WFD dosage. The CR degradation efficiency was promoted in the presence of chloride and sulfate ions and was inhibited in the presence of carbonate ions. The WFD was reused for three repeated processes, and the degradation performance of the CR decreased from 100 to 81.1%. The decreased CR degradation efficiency was due to the reduction of the active sites on the surface of WFD. This study provides useful insights into the environmental application of WFD from industrial waste.

### Supplementary Information

Below is the link to the electronic supplementary material.

Supplementary file1 (DOCX 472 KB)

## Data Availability

Data is available on the request to the corresponding author.
