# Supplementary material for: Congo red dye degradation using Fe-containing mineral as a reactive material derived from waste foundry dust
Source: Environ Sci Pollut Res Int. 2024 Mar 28;31(19):28443–53. doi: 10.1007/s11356-024-33064-9 (PMC11058770; doi:10.1007/s11356-024-33064-9)
Supplement: Supplementary file 1 — Supplementary file1 (DOCX 472 KB) [file 11356_2024_33064_MOESM1_ESM.docx]

**Figure S1** Calibration curve for Fe.

**Figure S2** Calibration curve for Congo red.

**Figure S3.** FT-IR of WFD before and after the reaction.


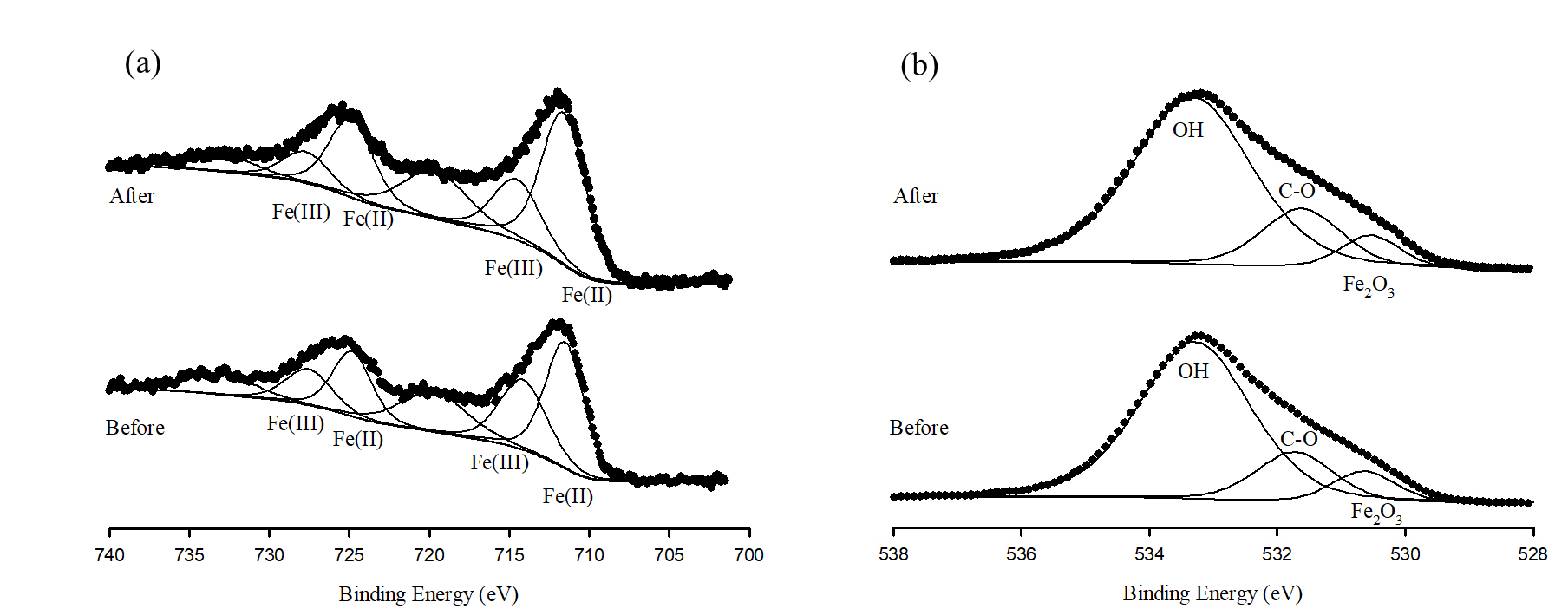


**Figure S4**. (a) Fe 2p and (b) O 1s high-resolution XPS spectra of WFD before and after degradation. The WFD catalyst after three cycles of degradation was used for characterization.
